# Isolated Prostate Tuberculosis Mimicking Prostate Cancer

**DOI:** 10.4314/ejhs.v34i1.10S

**Published:** 2024-10

**Authors:** Tesfaye Kebede Legesse, Semira Abrar Issa, Yodit Abraham Yaynishet, Tesfahun Amsal Dessie, Tewodros Yalew Gebremariam, Birhanu Kassie Reta

**Affiliations:** 1 Department of Radiology, Addis Ababa University, College of Health Sciences, Addis Ababa, Ethiopia; 2 Department of Surgery, Addis Ababa University, College of Health Sciences, Addis Ababa, Ethiopia; 3 Department of Pathology, Addis Ababa University, College of Health Sciences, Addis Ababa, Ethiopia

**Keywords:** prostate cancer, tuberculosis, prostate MRI, prostate biopsy, case report

## Abstract

**Background:**

Tuberculosis (TB) remains the deadliest infectious disease globally, with the kidneys being the most frequently affected organ in the genitourinary system. Isolated prostate involvement by tuberculosis is rare and may mimic prostate cancer. This case report aims to highlight the diagnostic challenges and therapeutic responses associated with isolated prostate tuberculosis, particularly in the context of significantly elevated prostate-specific antigen (PSA) levels in a TB-endemic region.

**Case Presentation:**

A 69-year-old male was referred to Tikur Anbessa Specialized Hospital (TASH) with obstructive lower urinary tract symptoms (LUTS) lasting seven months. He had previously undergone transurethral resection of the prostate (TURP), with histopathological findings suggestive of benign prostatic hyperplasia (BPH). Further investigation revealed an extremely elevated PSA level of 1768 ng/ml. Magnetic Resonance Imaging (MRI} raised high suspicion for locally advanced prostate cancer; however, a repeat biopsy and histopathology ultimately diagnosed TB prostatitis. The patient responded successfully to anti-tuberculosis therapy.

**Conclusion:**

Isolated prostate involvement by M. tuberculosis is rare, and extremely elevated PSA levels are unusual. Histopathological examination is a confirmatory test, but results can be falsely negative if a representative sample is not obtained. Therefore, a high degree of clinical suspicion, particularly in endemic areas, along with repeat biopsy, is crucial for accurate diagnosis.

## Introduction

Tuberculosis (TB) is a significant global health concern caused by *Mycobacterium tuberculosis*. While it primarily affects the lungs, extrapulmonary involvement is common, with the kidneys being the most frequently affected organ in the genitourinary system. Isolated prostate TB is exceedingly rare, accounting for only 2.6% of genitourinary TB cases. This condition often presents with mildly elevated PSA levels and imaging findings that may resemble those of advanced prostate cancer. Extremely elevated PSA levels in cases of prostate tuberculosis are exceptionally rare, leading to diagnostic challenges, especially in TB-endemic regions. This case report discusses an instance of isolated prostate tuberculosis presenting with an unusually elevated PSA level in a patient experiencing persistent LUTS for seven months.

## Case Presentation

A 69-year-old male was referred to Tikur Anbessa Specialized Hospital (TASH) in Ethiopia for obstructive LUTS. His medical history included a transurethral resection of the prostate (TURP) procedure, with results indicating BPH. During clinical examination, he reported difficulty urinating, hematuria, and frequent urination over a seven-month period. His International Prostate Symptom Score (IPSS) was 32. Notably, he exhibited no constitutional symptoms such as night sweats, fever, weight loss, or signs of chronic illness, making clinical suspicion for tuberculosis less likely. He had no personal or family history of tuberculosis or prostate carcinoma. Vital signs were stable, with a blood pressure of 110/80 mmHg, pulse rate of 84 beats/min, respiratory rate of 18 breaths/min, and temperature of 36.4°C. A digital rectal examination revealed an enlarged, firm prostate with smooth contours.

Further investigations included urine analysis, which showed sterile pyuria, and cystoscopy, which revealed moderately enlarged lateral and median lobes of the prostate without any urethral strictures. His PSA level was significantly elevated at 1768 ng/ml, raising suspicion for prostate cancer. An abdominal ultrasound confirmed an enlarged prostate measuring 89 ml without any apparent mass. Imaging of the kidneys, urinary bladder, seminal vesicles, and testicles was normal. Chest radiography and lumbosacral imaging revealed no abnormalities suggestive of tuberculosis or metastasis. Given the high PSA level, an MRI was performed for further investigation.

Pelvic MRI revealed an enlarged prostate gland with a volume of 95 ml. The transitional zone was enlarged with heterogeneous signal intensity on T2-weighted imaging (T2WI). A focal hypointense lesion was observed in the right basal and midzone of the prostate, extending into the peripheral zone, with capsular obliteration and extra-prostatic extension. This lesion demonstrated diffusion restriction on diffusion-weighted imaging (DWI) and apparent diffusion coefficient (ADC) mapping, leading to a PI-RADS score of 5. The urology team performed a transrectal biopsy using an 18-gauge core needle, which was initially reported as benign prostatic hypertrophy (BPH).

Despite the initial biopsy findings indicating BPH, the discrepancies between the elevated PSA level, MRI findings, and histopathological analysis necessitated a more definitive TURP and biopsy, as transrectal ultrasound scan (TRUS) was not available. Histologic sections showed areas of granulomatous inflammation with central caseating necrosis and multinucleated giant cells, surrounded by palisading epithelioid macrophages, lymphocytes, plasma cells, and fibroblasts. The remaining tissue consisted of nodular structures composed of variable-sized glands lined by basal and secretory cells embedded in fibromuscular stroma. No features of malignancy were noted, confirming tuberculous prostatitis alongside BPH.

The patient commenced anti-tuberculosis therapy for six months based on local guidelines. Combination therapy with rifampicin, isoniazid, pyrazinamide, and ethambutol was administered for two months, followed by isoniazid and rifampicin for four months. Follow-up indicated a significant reduction in the PSA level (83 ng/ml at two months), and the patient reported improvement in LUTS, with an IPSS score of 9. Unfortunately, the patient was lost to follow-up, preventing further PSA-level assessment.

## Discussion

Isolated prostate involvement is exceedingly rare, accounting for only 2.6% of genitourinary TB cases and seldom presenting with extremely elevated PSA levels, often leading to delayed diagnosis due to low clinical suspicion. (1) This case highlights the importance of considering tuberculosis in the differential diagnosis of prostate disorders, particularly in endemic areas, to prevent misdiagnosis and ensure timely and appropriate management.

Patients with prostate tuberculosis may present asymptomatically or with nonspecific lower urinary tract symptoms, complicating timely diagnosis and differentiation from prostate carcinoma. (1) A retrospective study described six clinical presentations of prostate TB, with the most common being symptoms resulting from prostatic obstruction, chronic prostatitis, and chronic epididymitis. (1) The spread of infection to the prostate is primarily hematogenous, although lymphatic spread, descending infections from the upper urinary tract, direct intra-canalicular extension, and post-intravesical BCG injections are also documented pathways.(2)

The serum PSA level can be mildly elevated in TB prostatitis; however, extremely high levels are typically associated with prostate cancer. The mechanism for elevated PSA levels in prostate cancer is primarily attributed to increased production and release from cancerous prostate cells. In contrast, tuberculosis is characterized by inflammation and necrosis, and the mechanisms leading to high PSA levels in prostate tuberculosis remain largely unexplained. One literature review suggested that the inflammation and necrosis associated with TB prostatitis may disrupt normal prostate architecture, releasing more PSA into circulation.(10) Although there was a case report of acute prostatitis with a PSA level of 1398 ng/ml, the reason for such an extremely elevated PSA level in that instance was not clarified.(3)

Urine analysis may reveal sterile pyuria, which should help clinicians make a differential diagnosis of tuberculous prostatitis. This patient also had sterile pyuria evidenced in his urine analysis. The identification of prostate tuberculosis on laboratory and imaging evidence can be challenging. The confirmatory diagnostic tests include histopathological examination and bacteriological evidence from culture.(2) More advanced and newer techniques for the diagnosis of prostate TB include cartridge-based nucleic acid amplification test (CBNAAT) of biopsy tissue and urine PCR. These techniques are applied when other diagnostic tests fail to detect mycobacteria.

Imaging findings can also be misleading and may mimic prostate cancer; however, a discordance between a low PIRAD score and the presence of invasive features in a lesion in the peripheral zone is found to be suspicious for granulomatous prostatitis. The patient in this case had a lesion involving both the peripheral and transitional zones, with a PI-RADS score of 5 due to the size, signal characteristics of the lesion, and extracapsular extension.

Two imaging patterns are suggested on MRI for prostate tuberculosis: the nodular pattern, characterized by several small nodules that are hypointense on T2-weighted imaging (T2WI) and show no significant diffusion restriction, and the diffuse pattern, in which the entire gland is affected by hypointense bands on T2WI, known as the watermelon skin sign. This pattern demonstrates diffusion restriction, which can be attributed to inflammatory cell infiltration in tuberculosis. (4)

Prostate tuberculosis typically affects both the peripheral and transitional zones as in this case. The T2WI hypointense signal and the enhancement characteristics mimic malignancy. In suspected tuberculous prostatitis, MR spectroscopy is said to add to the diagnostic efficacy in differentiating from cancer as prostate TB shows citrate levels in the normal range. (4)

MRI in this patient played a critical role in prompting further investigation and diagnosis, as the first biopsy was not representative of the patient's primary pathology. Transurethral resection of the prostate (TURP) and biopsy ultimately revealed the findings after a negative transrectal biopsy; however, the initial transrectal biopsy was performed without ultrasound guidance. This case highlights the importance of utilizing transrectal biopsy with ultrasound guidance to improve the efficacy of lesion detection, rather than relying on random biopsy methods.

The mainstay of management for TB prostatitis is medical treatment using multiple anti-TB drug combinations. Surgical therapy can be considered if patients do not respond to medical therapy or if a prostatic abscess is present, for which transurethral loop drainage is a feasible alternative option. PSA levels should be monitored in cases of elevated PSA to rule out an underlying prostate cancer. (5) In an extremely elevated PSA level, it was suggested to consider ongoing acute prostatitis as a possibility and repeat PSA level after treatment.(3) Isolated involvement of the prostate with M. tuberculosis is rare, and an extremely elevated PSA level, as seen in this case, is very unusual. Delays in diagnosis can lead to complications such as abscess formation, urethral stricture, and systemic dissemination of the disease, all of which may significantly impact a patient's quality of life and reproductive health. (2) One of the confirmatory diagnostic tests is histopathologic examination; however, the result can be falsely negative if a representative sample is not taken. As such, a high degree of clinical suspicion particularly in TB-endemic regions and repeat biopsy are crucial for establishing the diagnosis.

**Ethical Approval and Informed Consent:** Informed written consent was obtained from the patient for the publication of medical case details. All patient information has been anonymized to ensure confidentiality. No institutional approval was required for the publication of this case report.

## Figures and Tables

**Figure 1:**
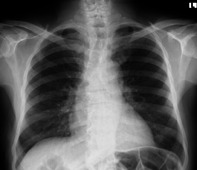
Frontal chest radiograph which was normal with no evidence of pulmonary tuberculosis or metastasis

**Figure 2:**
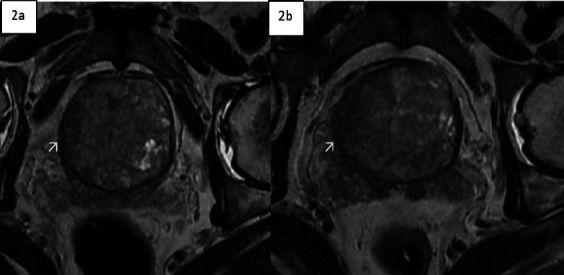
T2W axial MR images at different levels showing focal T2 hypo-intense lesion located in the right lateral side of an enlarged transitional zone of the prostate (2a shown with an arrow) and extension to the peripheral zone and areas of extra-prostatic extension (2b shown by the arrow

**Figure 3:**
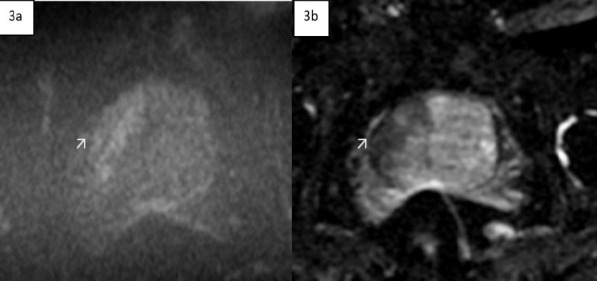
The lesion shows evidence of diffusion restriction on the DWI (3a) and ADC mapping 3b

**Figure 4:**
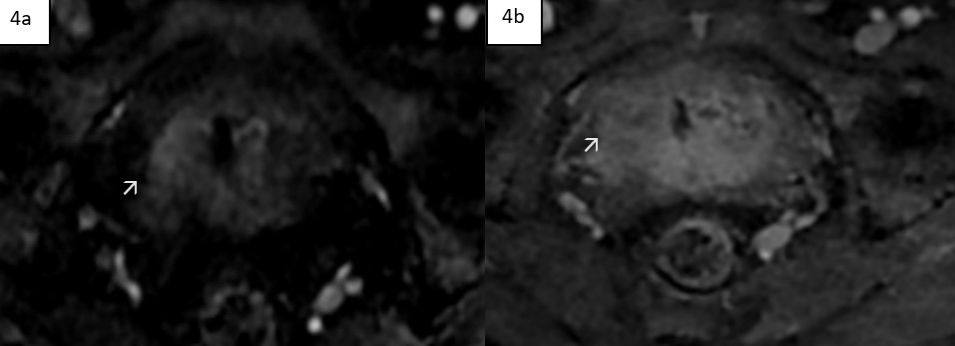
There is early contrast enhancement of the focal lesion on dynamic post contrast study (4a arrow) with relative progressive washout (shown in the arrow in 4b

**Figure 5:**
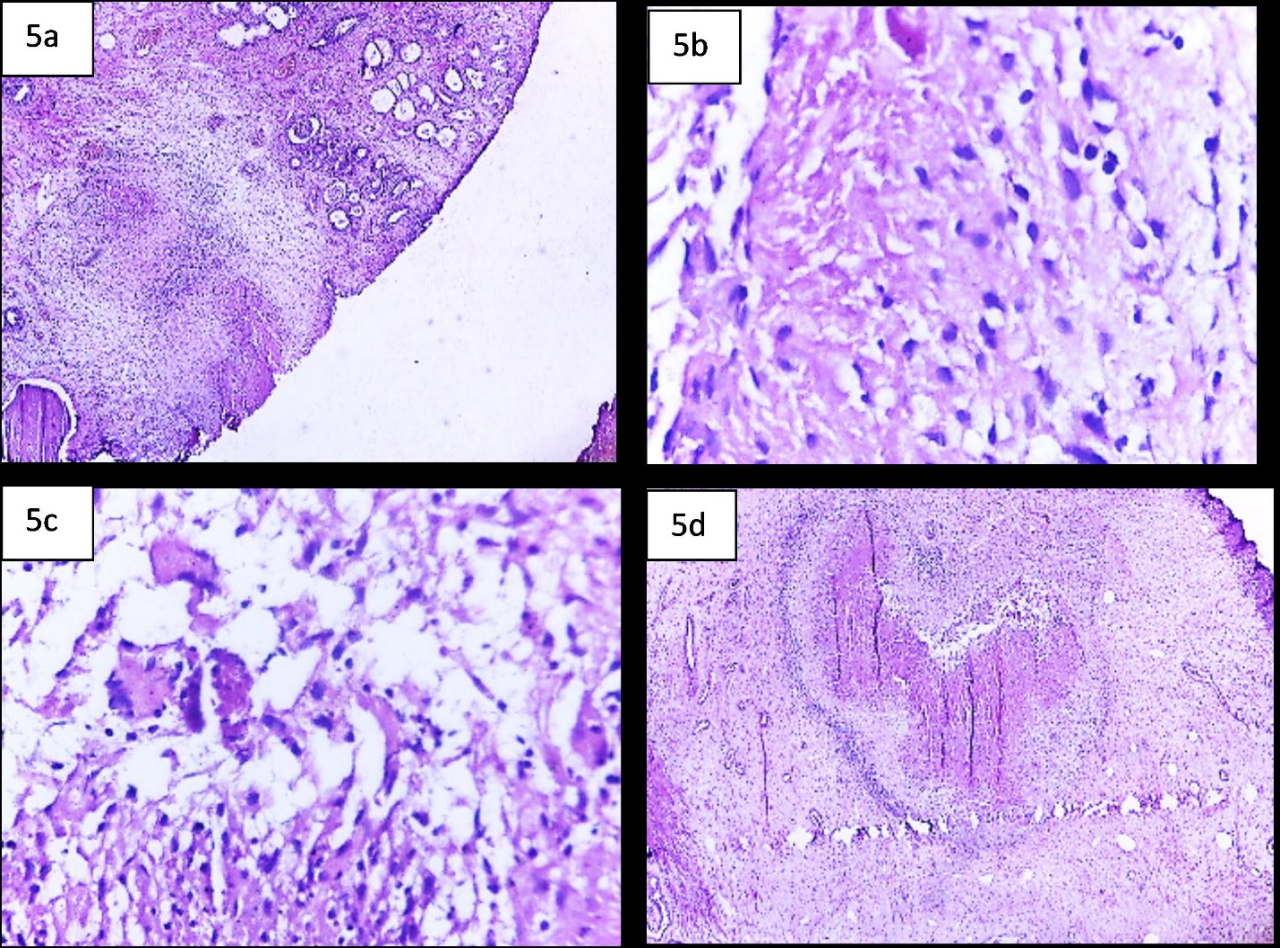
(a) bilayered prostatic glands and adjacent caseous granulomatous inflammation, (10X) b) necrosis and surrounding epithelioid histiocytes, (40X) c) multinucleated giant cells, (40X) d) necrosis surround by lymphoplasmacytic cells and histiocytes, (10X)

## References

[1] Mishra KG, Ahmad A, Singh G, Tiwari R (2019). Tuberculosis of the prostate gland masquerading prostate cancer; five cases experience at IGIMS. Urol Ann.

[2] Figueiredo AA, Lopes HE, Barreto AA, Fanni VSS, Bastos Netto JM (2024). Prostate Tuberculosis: six forms of clinical presentation. Int Braz J Urol.

[3] Nepal A, Sharma P, Bhattarai S, Mahajan Z, Sharma A, Sapkota A (2023). Extremely Elevated Prostate-Specific Antigen in Acute Prostatitis: A Case Report. Cureus.

[4] Cheng Y, Huang L, Zhang X, Ji Q, Shen WJKjor (2015). Multiparametric magnetic resonance imaging characteristics of prostate tuberculosis. Korean J Radiol.

[5] Tapsoba AK, Rahoui M, Bibi M, Chelly B, Ouanes Y, Chaker K (2021). An unusual association of adenocarcinoma and isolated tuberculosis of the prostate gland. J Surg Case Rep.

